# R-SPEAK, an AI-Based Smartphone App to Enhance Communication in People With Expressive Aphasia: Qualitative Acceptability and Usability Study

**DOI:** 10.2196/93518

**Published:** 2026-10-08

**Authors:** Abdel-Karim Al-Tamimi, Jacob Andrews, Jacqueline Benfield, Cath Sweby, Chris Gilmartin, Rebecca Lindley, Diane Trusson, Molly Dziunka, Dee Webster, Kathryn Radford

**Affiliations:** 1 School of Computing and Digital Technologies, Sheffield Hallam University, City Campus, Howard Street, Sheffield, England, S1 1WB, United Kingdom, 44 114 225 5555; 2 Computer Engineering Department, Yarmouk University, Irbid, Jordan; 3 Biomedical Research Centre, University of Nottingham, Nottingham, England, United Kingdom; 4 Derbyshire Community Health Services NHS Trust, Chesterfield, England, United Kingdom; 5 Centre for Rehabilitation and Ageing Research, University of Nottingham, Nottingham, England, United Kingdom; 6 Northern Care Alliance NHS Foundation Trust, Manchester, England, United Kingdom; 7 Nottingham University Hospitals NHS Trust, Nottingham, England, United Kingdom; 8 Mental Health and Clinical Neurosciences Academic Unit, University of Nottingham, Nottingham, England, United Kingdom; 9 Sheffield Teaching Hospitals NHS Foundation Trust, Sheffield, England, United Kingdom; 10 Health Sciences School, University of Sheffield, Sheffield, England, United Kingdom; 11 NIHR HealthTech Research Centre for Rehabilitation, University of Nottingham, Nottingham, England, United Kingdom

**Keywords:** aphasia, AAC, digital health, communication, health technology, GenAI, LLM, human-AI interaction, affective computing, large language model, augmentative and alternative communication, generative AI

## Abstract

**Background:**

Aphasia, an acquired language disorder that affects the ability to understand and produce language, significantly impairs effective communication. Large language models (LLMs) such as ChatGPT may help by generating fluent and coherent text, offering new ways to support communication for people with aphasia.

**Objective:**

This study aims to coproduce a communication support system using LLMs and evaluate its usefulness and acceptability among people with mild-to-moderate expressive aphasia.

**Methods:**

We used the Double Diamond approach across 3 phases. Phase 1: a stroke-survivor patient and public involvement (PPI) group (n=4) and the research team used MoSCoW prioritization (Must Have, Should Have, Could Have, Will Not Have) to rank ideas and co-design a software solution (Revolutionizing Speech Enhancement in Aphasia Using Knowledgeable-AI [R-SPEAK]) to augment verbal communication. Phase 2: 8 LLMs were evaluated on interpreting aphasic utterances from AphasiaBank transcripts. Outputs were compared, and the best responses, selected by team consensus, formed ground truth. The model producing the highest-quality responses was used in the prototype. Four people with aphasia and one carer (n=5) evaluated the prototype in semistructured interviews, and a health care professional (HCP) focus group (n=6) evaluated the concept and prototype. Topic guides were informed by the technology acceptance model (TAM), and thematic analysis themes were mapped onto its constructs. People with aphasia (n=4) rated prototype usability using the System Usability Scale (SUS). Phase 3: to improve processing speed, 12 lightweight LLMs (0.5-4 billion parameters) were evaluated on interpreting real aphasic speech using an LLM-as-a-judge framework to assess answer relevancy, faithfulness, and completeness.

**Results:**

In Phase 1, PPI groups (people living with aphasia, carers, and HCPs) identified essential system features and contextual data requirements across 2 co-ideation sessions using MoSCoW prioritization. In Phase 2, Mixtral 8x7B, the best-performing LLM for interpreting aphasic utterances, was used for the prototype. People living with aphasia rated R-SPEAK as good (SUS score: mean 75, SD 9.19). Themes mapped across three TAM constructs. (1) Attitude toward using it: people living with aphasia had high hopes, while clinicians were more cautious about its benefits. (2) Perceived ease of use: participants found it easy, though potentially harder for those with other poststroke impairments or more severe aphasia, with training possibly needed. (3) Perceived usefulness: R-SPEAK could be useful in many scenarios and improve independence. Recommendations included improved accuracy, speed, and tailored interface modifications. Phase 3 showed Qwen2.5:3B performed strongest overall, with high faithfulness and sub-second latency, while models under 1.5 billion parameters showed pronounced hallucination, indicating a lower bound on model capacity for reliable clinical speech interpretation.

**Conclusions:**

Our co-designed AI-supported R-SPEAK prototype was considered acceptable to patients. Next steps involve refining and developing a smartphone app for feasibility testing in a larger cohort of people with mild-to-moderate aphasia.

## Introduction

### Background

Aphasia is an acquired communication disorder and affects around 41% of UK stroke survivors [1]. It can also occur in other acquired neurological conditions such as brain tumors and frontotemporal dementias, particularly primary progressive aphasia. There is a broad phenotype of aphasia, where some individuals are unable to say single words or understand language at all, while others have milder difficulties, such as reduced sentence complexity or word-finding errors, which may be sound-based (phonological) or meaning-based (semantic) [2,3]. Speech production may be more effortful, with a reduction in speech rate, or with changes in intonation and prosody, leading to an overall reduction in verbal fluency and efficiency. Aphasia can affect both spoken and written language with wide-ranging, life-changing consequences [4]. All severities of aphasia can profoundly affect a person’s life, including their ability to access rehabilitation, engage in daily activities, maintain relationships, and participate in society, including returning to work and leisure [5]. This can often lead to social isolation, low mood, and reduced quality of life [6,7].

While rehabilitation is important to help people living with aphasia regain as much language as possible, individuals often continue to experience aphasia and its impact over the longer term and are faced with the challenge of learning to live with aphasia using their preserved language.

Augmentative and alternative communication (AAC) options, such as digital or paper-based word, picture, or letter grids for people with aphasia, aim to improve communication of basic needs [8]. These systems often have a limited vocabulary and require preprogramming by a clinician or carer. Furthermore, the purpose of communication is much more than expressing basic needs, and people with communication difficulties are more dependent on coconstructed interactions with a communication partner [9].

AI may offer a solution and has been used to augment nonimpaired speech in several ways, including predictive texting [10], speech-to-text technologies [11], language translation and interpretation [12], speech synthesis [13], and voice cloning [14].

Text-to-speech (TTS) technologies draw on recent advances in natural language processing (NLP) techniques, and the introduction of large language models (LLMs) such as Google Gemini, OpenAI’s GPT-3/4, and Meta’s Llama. LLMs operate on user-provided prompts and can generate coherent paragraphs of text [15] and predict and generate human-like sentences by learning from vast textual datasets. While LLMs differ in efficiency and accuracy, prompt engineering (the art of crafting effective prompts) can yield varying results [16].

Aphasia often includes agraphia (written language impairment) and cannot use TTS technologies to augment their communication [17]. By incorporating automatic speech recognition (ASR) technology into these systems, this approach enables the removal of barriers to accessing technology for those with communication difficulties and supports the development of personalized AAC devices to support communication. However, ASR systems have been trained on a largely nonimpaired speech dataset, and accuracy in interpreting impaired speech is much lower [18]. Much work is being done to improve ASR for dysarthric speech (motor speech disorder) by training LLMs with disordered speech [19], but this has not been explored for people with linguistic impairments such as aphasia.

Moreover, AI’s ability to learn from the daily activities and contextual data of the individual with aphasia means that it can offer bespoke solutions tailored to the individual’s needs. By learning the user’s behavior, mistakes, and preferences, these tools can adapt to offer more accurate or efficient communication support over time.

Leveraging LLMs in this way offers the potential to reconstruct aphasic speech and translate it into spoken language, improving comprehensibility and communication. As demonstrated in Figure 1, our proposed “Revolutionizing Speech Enhancement in Aphasia Using Knowledgeable-AI” (R-SPEAK) technology has the potential to support clearer communication, helping the person with aphasia regain control over their personal communication interactions, increasing autonomy, reducing social isolation, and improving their quality of life. It has the potential to revolutionize the lives of people living with aphasia, caregivers, and health care professionals (HCPs) involved in their rehabilitation.

**Figure 1.**
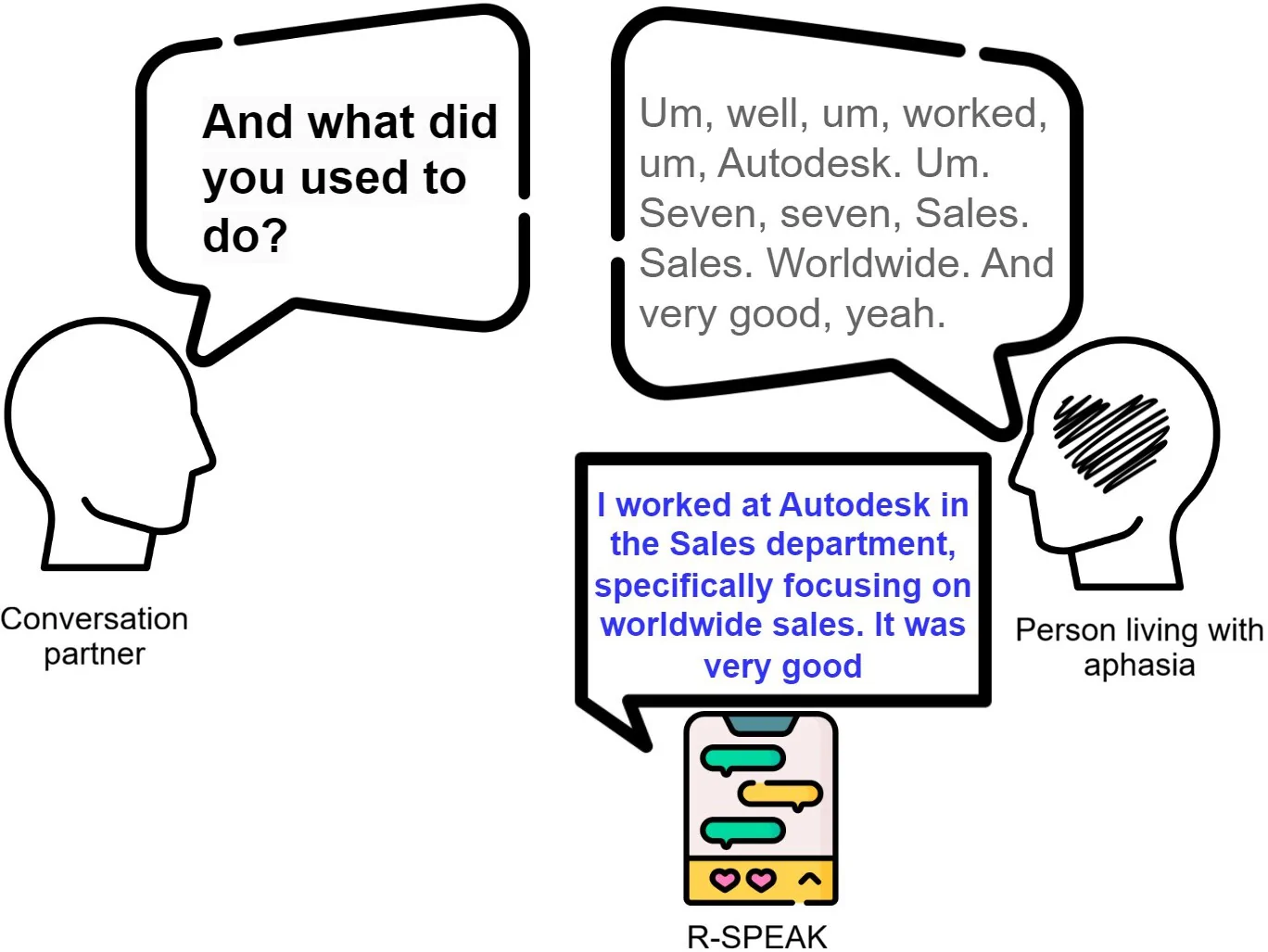
Example of how Revolutionizing Speech Enhancement in Aphasia Using Knowledgeable-AI (R-SPEAK) converts real-world aphasic speech into comprehensible speech.

### Aim

Working with people with aphasia and stroke clinicians, we aimed to co-design and evaluate a prototype for a portable device (eg, mobile device) that incorporates LLMs to enhance the clarity and fluency of verbal expression of people living with mild-to-moderate expressive aphasia.

### Study Objectives

The study is structured around three core objectives that collectively guide the co‑design, technical evaluation, and real‑world implementation assessment of our proposed AI‑supported communication device (R-SPEAK).

- To co-design and develop a prototype of an AI-supported portable device with people with aphasia and stroke clinicians that incorporates LLMs to enhance the clarity and fluency of verbal expression in mild-to-moderate expressive aphasia.
- To evaluate the technical effectiveness and usability of the prototype device through testing with people with aphasia in real-world communication scenarios, assessing its impact on communication clarity and fluency.
- To assess the acceptability, perceived utility, and implementation feasibility of the device among people with aphasia and health care workers, including identification of barriers and facilitators for adoption.

## Methods

### Overview

This study used a mixed methods participatory design approach using the Double Diamond methodology to co-design and evaluate a prototype of a communication support device for people with mild-to-moderate expressive aphasia [20]. A detailed, phase-by-phase account of the design is provided in the “Design” section below. Phase 2 (Develop and Demonstrate) comprised iterative technical development of the R-SPEAK prototype and its evaluation through usability testing with people with aphasia (n=5) and an HCP focus group (n=6). Phase 3 (Refine and Redesign) involved a systematic evaluation of lightweight open-weight LLMs to improve processing speed (see the “Results” section).

### Concept

Our methodology capitalizes on the recent advancements in NLP, particularly the emergence of LLMs, to enhance the speech comprehension of individuals living with aphasia. Our concept is for a system that uses LLMs’ language-understanding capabilities to build upon the utterances of people with mild-to-moderate aphasia to produce coherent speech. The process begins by recording the speech of people living with aphasia and converting it into text using speech-to-text or ASR technology, as shown in Figure 2. We use these textual data and the speech of the conversation partner or interlocutor to craft precise prompts for LLMs.

**Figure 2.**
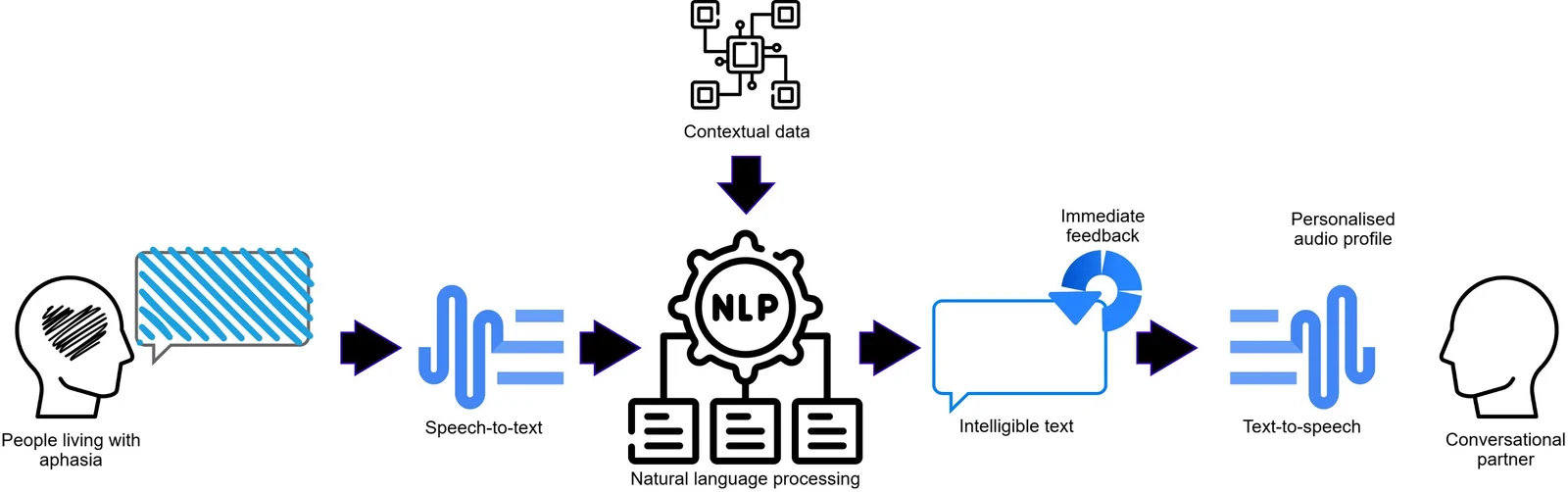
Overview of the Revolutionizing Speech Enhancement in Aphasia Using Knowledgeable-AI (R-SPEAK) system workflow for enhancing communication in people with aphasia. NLP: natural language processing.

The LLM analyzes the prompt-provided data, endeavoring to generate coherent interpretations of the speech by completing missing words, inferring unclear terms, and eliminating unnecessary filler words. The generated text is presented to the user, who selects the most suitable transformation that effectively conveys their intended meaning, with the option to edit and manually adjust the text as needed. Subsequently, the chosen text is transformed into spoken words using TTS technology, with the option to use a synthesized voice personalized to the patient’s audio profile. The resulting clear, coherent sentences can be easily understood by the person’s conversation partner. The modular design of our system provides several key advantages: it allows for personalization to individual user needs, can continuously improve as language models advance, integrates well with existing assistive technologies, and focuses on locally deployable LLMs with the option to access cloud-based LLMs through internet connectivity when needed.

### Design

Our research methodology used the Double Diamond design approach, a systematic framework that structures the design process into 4 stages across 2 phases, as shown in Figure 3 [20]. Phase 1 encompassed the Discover and Define stages, systematically exploring communication challenges faced by people with aphasia and identifying specific user needs through patient and public involvement (PPI). This involved divergent exploration of the problem space followed by convergent synthesis to define clear design requirements.

**Figure 3.**
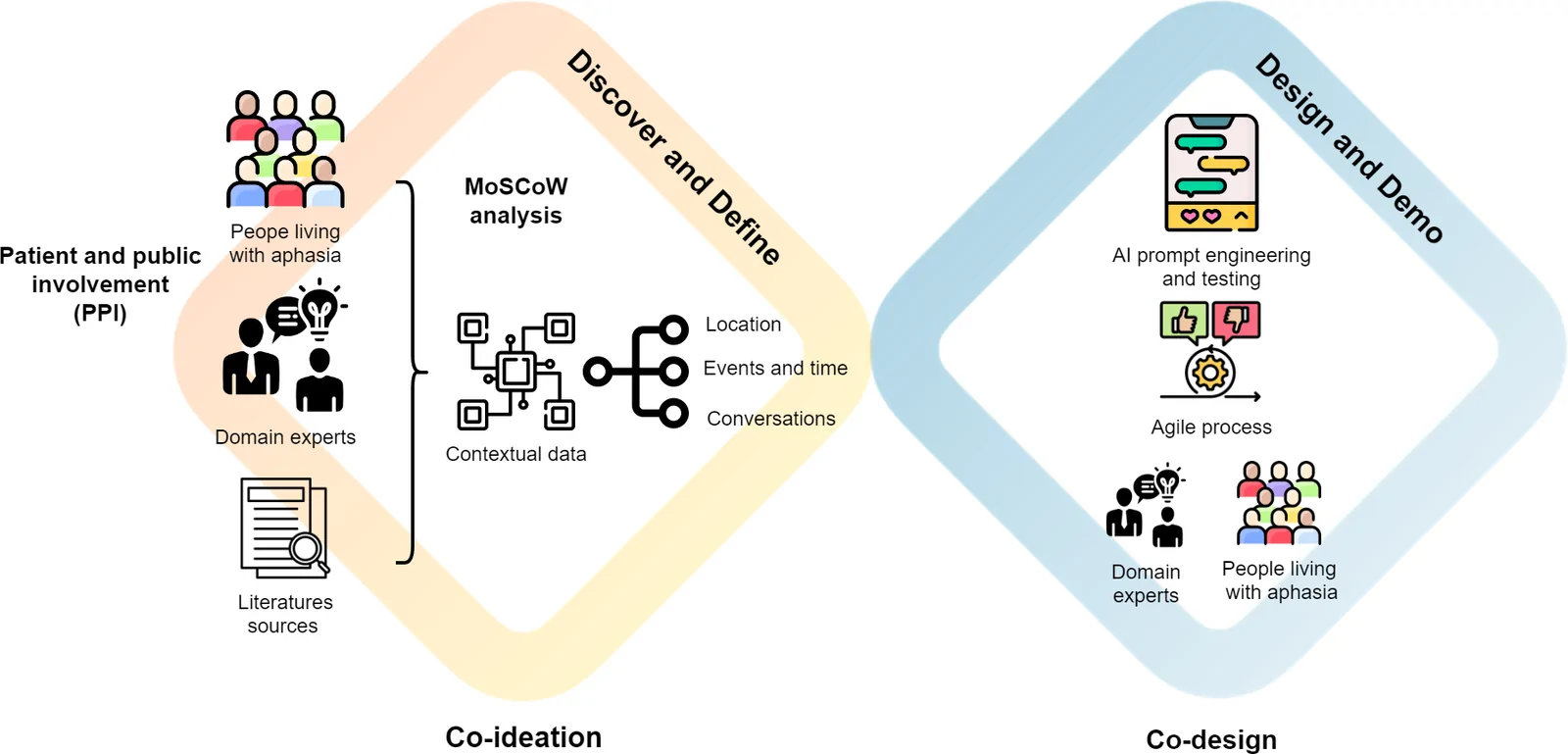
Double Diamond approach to prototype development.

Phase 2 comprised the Develop and Demonstrate stages, focusing on iterative development of the R-SPEAK technological solution and subsequent validation through user testing. Throughout both phases, participatory co-design principles ensured meaningful involvement of people with aphasia and stroke clinicians through workshops, interviews, and focus groups conducted across multiple iterative stages. This collaborative approach ensured the technological solution remained grounded in real-world user needs and clinical practice requirements. All qualitative findings are reported in accordance with the COREQ (Consolidated Criteria for Reporting Qualitative Research) [21] checklist to ensure methodological transparency and rigor.

### Phase 1: Discover and Define

#### Overview

As part of the “Discover and Define” phase of the Double Diamond design process, PPI was integral to identifying communication challenges and clarifying user needs.

#### PPI

##### Overview

We convened a PPI group consisting of people with experience of living with aphasia and their carers. We recruited individuals with aphasia through established networks, including members of the research team’s existing contacts and participants from stroke survivor groups such as the Nottingham Stroke Partnership Group and Speakeasy (Stockport), following an initial presentation and invitation to participate. The Phase 1 ideation group comprised 3 stroke survivors and 1 carer (n=4).

We involved the PPI group in several ways: (1) to inform system development; (2) to support recruiting for interviews to evaluate the system for usability, accessibility, acceptability, and usefulness; and (3) to review participant-facing materials to ensure accessibility for people with aphasia before use.

We held 2 online PPI group meetings: one with people with aphasia and their carers, and another with the research team, who were also HCPs with experience of working with people living with aphasia (KR, JA, CS, CG, and JB). During the meetings, we presented the concept and specific design options both verbally and with simple graphics. We used yes or no, closed, and open questions to facilitate discussion and gather feedback on the proposed system.

##### Co-Ideation

We used MoSCoW prioritization (Must Have, Should Have, Could Have, Will Not Have) [22] to systematically evaluate ideas and prioritize the features most critical to people with aphasia. This approach guided our understanding of which elements were essential for ensuring usability, accessibility, acceptability, and practical utility in real-world contexts. Through this structured process, we also identified the types of contextual data required to enhance the accuracy and relevance of the app’s outputs.

To facilitate effective communication and engagement with participants, we used visual aids such as images, keywords, and simplified infographics to help convey complex ideas. Group sessions were led by facilitators experienced in working with individuals with aphasia, who ensured sessions were inclusive by allowing extended response times and using yes or no or closed-ended questions to accommodate expressive language difficulties.

### Phase 2: Design and Demo

#### App Design (System Frontend)

We concentrated our efforts on designing an intuitive user interface and refining the underlying prompt engineering approach. Our computer scientist (AA) developed a low-fidelity prototype of the app’s frontend using Proto.io, a flexible prototyping platform well-suited for rapid iteration. This prototype was informed by insights gathered through engagement with PPI groups and close collaboration with clinical team members. Their feedback played a critical role in shaping both the visual layout and functional requirements of the interface, ensuring that it is user-friendly, accessible, and aligned with real-world needs in clinical settings.

#### Technical Development (System Backend)

To develop a functional prototype capable of reconstructing intelligible speech, one team member (MD) extracted 30 stand-alone question-and-answer pairs from AphasiaBank [23,24], a shared multimedia database of audio- and video-recorded conversations with individuals living with aphasia that is widely used in aphasiology research. The pairs were selected to represent everyday conversational exchanges (eg, questions about daily activities, preferences, and personal experiences) produced by speakers with mild-to-moderate aphasia, to reflect the type of input the prototype would need to interpret in real-world use. The selected dialogue pairs were converted from the Codes for the Human Analysis of Transcripts (CHAT) format [23] into plain text using a bespoke conversion tool developed by (AA).

These transcribed question-response pairs were then processed using 8 open-weight, locally deployable LLMs: Mixtral:8x7B, Gemma2:9B, Qwen2.5:7B, Llama3.1:8B, Phi-3:7B, WizardLM:7B, Mistral:7B, and Llama2:7B. These models were chosen based on their advanced reasoning capabilities and demonstrated adherence to user instructions. Several zero-shot prompting strategies were tested, in which the prompts included contextual information about aphasia and the characteristic differences between aphasic and typical speech patterns.

The outputs generated by the LLMs were independently evaluated by 5 members of the research team (CG, JA, JB, RL, and AA), all with clinical and/or technical expertise in LLMs. Evaluators assessed each LLM’s ability to interpret and reconstruct the intended meaning behind the original aphasic responses. For each question-answer pair, the team selected the most accurate LLM-generated response. Based on this expert consensus, the highest-performing LLM, in this case Mixtral, was identified and subsequently integrated into the development of the R-SPEAK prototype.

### Interviews and Focus Groups

#### Development of the Topic Guide

The aim of the qualitative interviews and focus groups was to understand HCPs’ and people with aphasia’s views and perceptions of the R-SPEAK prototype, to gather insights on its functionality, and to understand potential benefits and challenges. Two topic guides informed by the technology acceptance model (TAM) [25] were developed to address the different perspectives of HCPs and people with aphasia. Developed by Davis [25], the TAM is used to shed light on the processes involved in the acceptance of technology. TAM suggests that attitudes toward using innovative technology are based on perceptions of both usefulness and ease of use [26].

The topic guides were developed by RL, a female rehabilitation psychology researcher with extensive qualitative research experience with people with stroke and HCPs. The guides were reviewed and edited based on feedback from the wider research team with speech and language therapist (SLT) expertise (JB), extensive qualitative research experience (KR and JA), as well as expertise related to AI (AA).

#### Recruitment

Recruitment took place between July and August 2024, with interviews and the focus group conducted in September 2024.

#### Interviews

People with aphasia (all severities) were recruited via known contacts of the research team and aphasia support groups (Speakeasy), using convenience and snowball sampling [27]. Known contacts were approached by email or phone, and an aphasia-friendly recruitment poster was shared with potential participants in aphasia support groups, inviting them (or their carers) to contact the research team if interested. Those expressing interest in the interviews were sent an information sheet and consent form by post or email, depending on preference, and asked to complete and return the consent form (using a Microsoft form if online, or using a [provided] stamped addressed envelope if via post) within 2 weeks of receiving the information sheet. Eligibility criteria were inclusive: self-reported diagnosis of stroke with difficulty speaking and able to give informed consent. Participants were offered an opportunity to meet, either in-person or online, with a member of the research team, to have the information sheet and consent form read aloud with aphasia-friendly pictorial participant information to assist understanding, and completion of the consent form where informed consent was demonstrated.

#### HCP Focus Group

Stroke clinicians were recruited through the research networks of the members of the research team and were invited to take part in a focus group exploring the practicalities and potential of the technology we are developing. They were eligible to take part if they were an SLT or another HCP with experience of working with people with aphasia. Those expressing an interest were sent a participant information sheet explaining that we had developed “some technology to help people with aphasia who have difficulty communicating, to speak clearly” and that we would show them an early prototype and ask them some questions about it, including how easy it is to use, how useful it is, and their opinions on the design. Informed consent was collected using an online consent form.

### Materials

To give participants an understanding of the tool, an aphasia-friendly Microsoft PowerPoint presentation was prepared for the interviews and focus groups. For the interviews with people living with aphasia, the web-based R-SPEAK prototype allowed it to be shared on the participants’ screen during the interviews. The use case for exploration was booking a holiday (ie, we aimed to test whether the system could be helpful in this use case). It consisted of 8 preprogrammed questions that had been co-designed by the research team and PPI group members and included questions such as “Where would you like to go on holiday?” When the participant was ready, they could use their mouse to click to start recording while they answered and click again to stop. The prototype then produced an output, displayed on the screen to the interviewer and interviewee.

The System Usability Scale (SUS) was used for objectively measuring perceptions of usability [28]. It consists of 10 statements such as “I think I would like to use this system frequently” and 5 response options from strongly disagree to strongly agree. Scores more than 70 demonstrate system acceptability. A slide deck was prepared in advance with one question per slide for sharing with the people living with aphasia who participated in the interviews.

### Procedure

Semistructured interviews and the focus group were conducted by an SLT and postdoctoral fellow (JB) and the research fellow (RL) via Microsoft Teams (Microsoft Corp) or Zoom (Zoom Communications, Inc). In the case of the interviews, participants were offered in-person interviews in their own homes if preferred. Potential participants were advised that a carer or an SLT could attend to support their communication.

At the beginning of both the interviews and focus group, the participants were introduced to the interviewers and shown the introduction presentation with a demonstration of R-SPEAK. Participants were asked a series of semistructured questions on usability, accessibility, acceptability, and usefulness of the system in line with the TAM [25].

Qualitative data from interviews and the focus group were audio-recorded, transcribed verbatim, and analyzed using a framework-guided thematic analysis informed by the TAM. Two researchers independently coded transcripts, generating initial codes inductively before mapping them to TAM constructs. Coding discrepancies were resolved through discussion, and themes were refined iteratively until consensus was reached. Representative quotations were selected to illustrate each theme.

In addition, during the interviews, the participants tested the prototype by answering the prepared questions and receiving the LLM-generated output for the 8 questions. To better understand the prototype in relation to the target population, participants were asked about their communication difficulties and experience of using technology and communication apps. Participants’ aphasia severity was rated by an SLT (JB) by listening to the interview recording and applying the Aphasia Severity Rating scale (Figure 4) [29], which enables a score of 0, 1, 2, 3, or 4 to be assigned, where 0=no functional communication and 4=very mild impairment that may be undetectable to the listener. At the end of the interview, participants were asked to rate prototype usability using the SUS.

**Figure 4.**
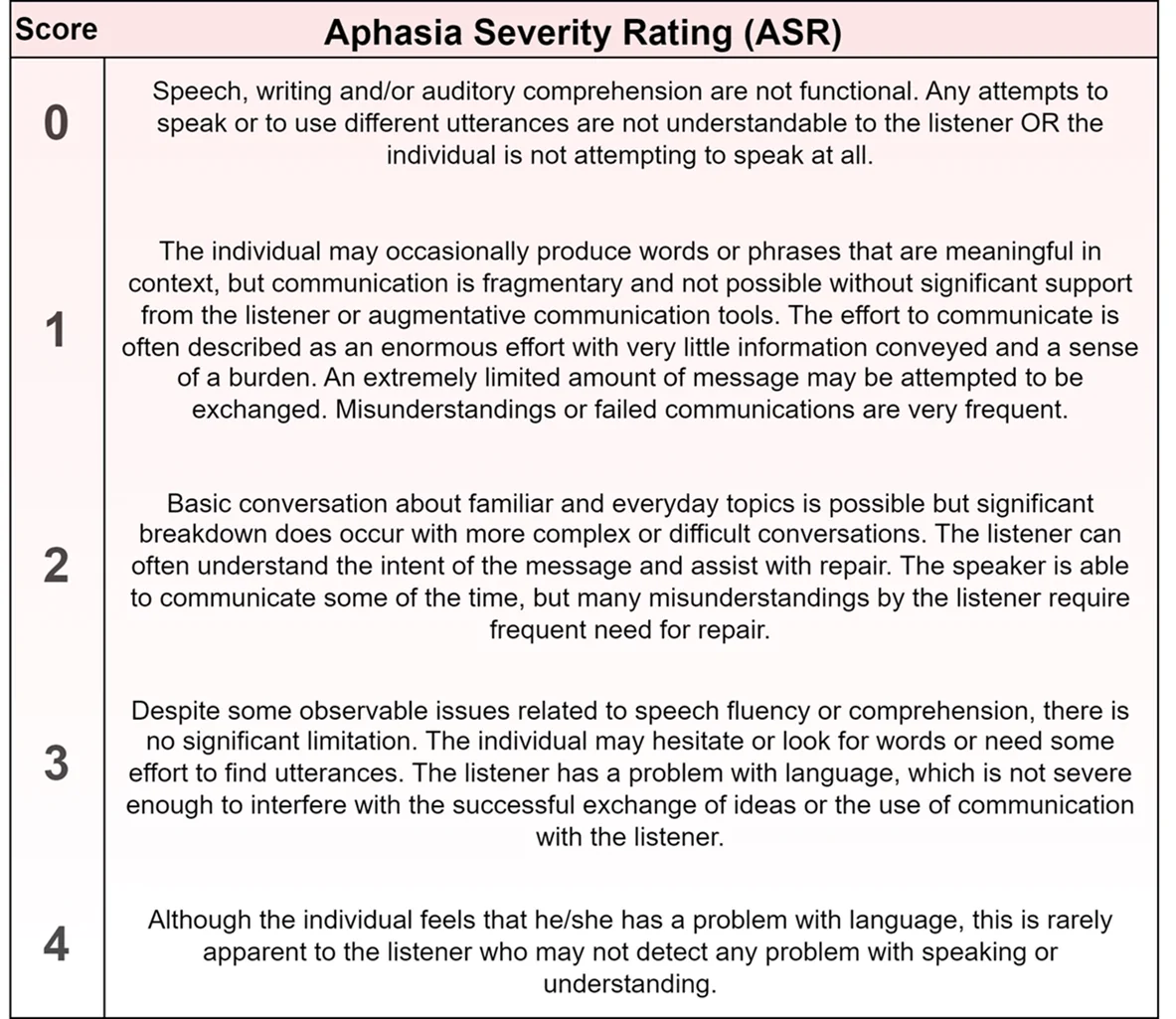
Aphasia Severity Rating (ASR) scale. This scale provides a structured index of aphasia severity, ranging from complete language impairment (0) to minimal or undetectable difficulties (4), based on speech, writing, and comprehension abilities. Adapted from Simmons-Mackie et al [29].

### Ethical Considerations

The study was approved by the Research Ethics Committee at Sheffield Hallam University (approval number ER64821246). All participants provided written informed consent before taking part. Interview participants were paid £50.00 for taking part. At the time of the study, £50 was equivalent to approximately US $63.68. The conversion was calculated using the prevailing exchange rate of 1.2736 GBP-USD.

### Data Analysis

The SUS raw scores were summed and converted into scores out of 100 as described by Lewis [28]. Individual and mean scores across the group are presented visually. Qualitative data from interviews and focus groups were recorded in either .mp3, .wav, or .mp4 format using encrypted computers owned by the University of Nottingham, transcribed verbatim, and analyzed using NVivo (Lumivero) and a framework-guided thematic analysis informed by the TAM. They were then transcribed and analyzed independently by 2 postdoctoral researchers (JB and DT) using NVivo and an inductive approach of data familiarization and development of themes [30,31]. The researchers then met to compare coding decisions, resolve discrepancies through discussion, and agree on code definitions. These discussions informed the development of a codebook containing code names, definitions, inclusion and exclusion criteria, and illustrative quotations. This was applied to subsequent transcripts and refined iteratively as new concepts emerged from the data. Once all transcripts had been coded, related codes were grouped into themes, which were refined iteratively until consensus was reached.

To enhance reliability, themes were discussed and aligned into one combined construct before being mapped onto the TAM constructs (ie, attitude toward using technology, perceived ease of use, and perceived usefulness) [25]. Representative quotations were selected to illustrate each theme. Given the nature of the participants’ communication impairments, transcripts were not returned to participants for checking. Recordings and anonymized transcripts were stored on a secure University of Nottingham password-protected server.

Both interviewers were female. RL had no prior relationships with the interview participants. However, some of the HCP participants in the focus group were known to JB. As the study was intentionally small and exploratory, it was not anticipated to achieve thematic saturation. However, the targeted inclusion of participants with highly relevant expertise within the sample is consistent with the concept of information power [32].

### Phase 3: Refine and Redesign

To improve the speed of processing in converting input to output, we conducted further experiments with open-weight lightweight LLMs. Open-weight LLMs are AI models where the trained parameters (weights) are publicly accessible, enabling organizations to download, run, fine-tune, and deploy these models on their own infrastructure. In these experiments, we aimed to reduce response time while preserving response relevancy, faithfulness, and completeness.

A total of 12 lightweight LLMs, with parameter sizes ranging from 0.5 to 4 billion, were evaluated using a dataset of 30 clinician-patient dialogue samples from individuals with poststroke aphasia. The lightweight models evaluated included Qwen2.5 (0.5B, 1.5B, and 3B), Hermes3 (3B), Gemma3 (1B and 4B), Phi3 (3.8B), Phi4-Mini (3.8B), Llama3.2 (1B and 3B), SmolLM2 (1.7B), and DeepScaler (1.5B). To account for stochastic generation variability, the generation protocol required each model to produce interpretations across 10 independent decoding iterations per sample, using a temperature setting of 0.7. This protocol resulted in an evaluation corpus of 3600 generated responses.

Model performance was systematically measured across 3 clinically grounded dimensions: answer relevancy (alignment with the original question), faithfulness (absence of hallucinations relative to the aphasic utterance), and completeness (coverage of key intent elements established by expert-curated reference interpretations). An automated LLM-as-a-judge framework using a larger language model (Qwen3:8B) was deployed to score each output on a continuous [0, 1] scale, with a minimum pass threshold set at 0.5 for all metrics. Finally, to assess practical deployability, inference latency was recorded in seconds for every individual generation.

To validate the LLM-as-a-judge framework, Qwen3:8B ratings were compared with a subset of human expert assessments and tested for consistency across repeated evaluations. This approach is supported by emerging evidence that larger language models can reliably assess short, structured outputs [33].

## Results

### Phase 1: Discover and Define

Three stroke survivors and one carer (n=4) were co-opted to the R-SPEAK PPI group and were involved in the co-ideation group during app development. While the MoSCoW prioritization exercise was instrumental in identifying key features for R-SPEAK, this study did not explicitly incorporate the would-not-have component of the framework. The research team’s focus during Phase 1 was on comprehensively identifying and cataloging potential features necessary for an effective communication support tool, prioritizing a broader scope over strict feature exclusion.

Based on this process, the PPI group identified and ranked the features considered most important for R-SPEAK. Must-haves: display the corrected phrase for user review, read it aloud, include a button to flag incorrect outputs, and allow private versus public conversations to prevent private data from informing future interactions. Should-haves: show the conversation partner’s message and allow selection of context or location (eg, doctor’s surgery). Could-haves: enable drafting and editing phrases in advance and provide suggested phrases for different contexts. Participants also raised concerns that people with more severe expressive or reading difficulties, or limited familiarity with technology, may struggle to use the app and its features. The output of this exercise subsequently informed prototype development in Phase 2.

### Phase 2: Develop and Demo

#### App Design and Technical Development

The LLMs were rated by 5 mixed experts. Mistral and Llama2 were excluded from consideration early due to their poor performance on basic language testing. Each expert completed an independent scoring sheet. Scores were normalized and discussed in a consensus meeting to reconcile differences. No formal interrater reliability metric was applied due to the exploratory nature of the evaluation. Mixtral outperformed all other LLMs, followed by Gemma and then the lightweight Qwen (Table 1). This consensus highlights Mixtral as the most effective model in interpreting responses from individuals living with aphasia among tested models.

**Table 1.** Expert evaluation of LLM^a^ performance through consensus and aggregate voting^b^.

Model	Consensus of votes (%)	Percentage of all votes (%)
Mixtral	36.7	40.4
Gemma	26.7	15.4
Qwen	20	25
Llama3	10	11.5
Wizard	6.7	7.7

a LLM: large language model.

b Consensus votes represent cases where all 5 experts unanimously selected the same model as superior. Percentage of all votes shows the distribution across all individual expert votes.

#### Interviews

Four stroke survivors with aphasia and one carer (n=5) were recruited for prototype testing and interviews. Three participants living with aphasia were assessed as having moderate aphasia, with one having milder impairment on the Aphasia Severity Rating scale. Characteristics of participants can be found in Table 2.

All had some previous experience with technology, including computers and smartphones, which they used for emails and online meetings, but none of the group used AAC apps or had previous experience with AI.

**Table 2.** Characteristics of interview participants.

Participant (P) ID	Self-reported difficulties with communication	Ability to use technology	Experience with AAC^a^ communication apps	Aphasia Severity Rating (by SLT^b^)
P1	Word-finding difficulties and reading and writing difficulties with longer text	Smartphone and computer for short emails, texts, and video calls	None	3 - mild
P2	Able to communicate but talking is difficult, especially numbers. Reading is okay	Computer and smartphone	Used apps for speech therapy exercises	2 - moderate
P3	Difficulties getting words out and reading more than single sentences	Computer, iPad, and smartphone and uses for online meetings, accounts, and emails	None	2 - moderate
P4 and carer 1	Reduced fluency, difficulty getting out words and reading, often missing key words	Has a smartphone and laptop. Needs support with emails, getting onto video calls	None	2 - moderate

a AAC: augmentative and alternative communication.

b SLT: speech and language therapist.

#### Focus Group

Five SLTs and one physiotherapist (n=6) were recruited for the focus group. All members had experience of working with people who had aphasia and across different clinical settings. Table 3 details their experience.

Framework-guided thematic analysis identified similarities and differences in perceptions of R-SPEAK among people with aphasia, carers, and HCPs. Themes are presented under the constructs of the TAM framework [25]; themes are summarized in Textbox 1.

**Table 3.** Characteristics of HCP^a^ focus group participants.

Participant	Details
SLT^b^ 1	Works in a neurorehabilitation center with patients with acquired brain injury.
SLT 2	Advanced clinical practitioner. Stroke-specific. Lots of patients with aphasia every day.
SLT 3	Works in a community neurorehabilitation team with patients with acquired brain injury and stroke.
SLT 4	Works in stroke rehabilitation. Regularly sees people with aphasia, dysarthria, and apraxia of speech.
SLT 5	Works in stroke rehabilitation wards. Worked with a lot of people who have aphasia.
PT^c^ 1	Works with SLT5 on acute and chronic stroke and outpatient wards.

a HCP: health care professional.

b SLT: speech and language therapist.

c PT: physiotherapist.

Textbox 1. **Themes from interviews and focus group mapped onto technology acceptance model constructs.**

**Attitude toward using**

- High hopes versus skepticism or somewhere in between
  - People living with aphasia and carers had high hopes
  - Clinicians more skeptical
  - Cautious optimism
- False assumptions about using AI
- Cost could be prohibitive

**Perceived ease of use**

- Dependent on type and severity of aphasia
- Other neurological impairments may pose challenges
- Tech easy for some
- Try before you buy
- Some training needed

**Perceived usefulness**

- Improved independence
- Could be useful in many scenarios

### Attitudes Toward Using R-SPEAK

#### High Hopes Versus Skepticism or Somewhere in Between

Overall attitudes varied between the people living with aphasia and HCPs. People living with aphasia and carers had high hopes that this was a life-changing technology, but clinicians were more skeptical about it being groundbreaking

*I think it’s marvellous really. Yeah, really good.*

[P2]

*I'm really excited about this, aren't you?*

[C1]

*Don’t want to give people false hope, thinking that this is going to solve everything, because actually conversation has so many more layers to it than just the language. So I’m a bit worried about people thinking that it’s going to solve things.*

[SLT3]

There was cautious optimism by both groups; HCPs suggested that it could be good to have another tool in the toolbox and may be less stigmatized than other AAC aids in use, but both groups want to see R-SPEAK develop and be used in action to make their minds up about it.

*I don’t think I I’ve got enough knowledge from it to say whether it would be really good or yeah, because I think it needs to some any because that’s a prototype and it only one scenario and it wasn’t very long.*

[P1]

*People might be more willing to use it than things that look potentially stigmatised, like some of the older AACs*

[SLT4]

*It’s sort of keeping a bit open to trying it, but not presenting it as an answer to aphasia. So we might see it as one more tool that would work with some people.*

[SLT4]

#### False Assumptions About AI

HCPs expressed concern about using AI in health care apps but specifically about the security of personal information of people living with aphasia. Some people living with aphasia might not be able to make informed choices about using the technology if it is based on reading data-sharing agreement text.

*AI in general has got a bit of controversy as well. So I also wonder if that might be a barrier to some people engaging.*

[SLT1]

However, the people living with aphasia we interviewed were not concerned at all.

*In terms of sort of using the AI of in this sort of yeah, I think it would be OK. But but sort of having an app on your phone that you can control that I think it’s OK. Yeah, yeah, yeah, I can turn it off anyway.*

[P1]

#### Costs Could Be Prohibitive

HCPs voiced concerns about costs to people living with aphasia given their experience of other apps and software options. However, this was not raised as a concern by people living with aphasia who we interviewed.

*Do you pay a tenner and you have it forever? Or are you having to pay like £15 a month forever? [...] obviously the more it’s charging, the more inequality we get with it.*

[SLT4]

### Perceived Ease of Use of R-SPEAK

#### Dependent on Type and Severity of Aphasia

Both people living with aphasia and HCPs acknowledged that there may be people who would find R-SPEAK more difficult to use or who would not be able to use it at all due to their aphasia. They gave specific examples, such as people who were unable to read or read quickly enough, people with auditory comprehension difficulties, those with jargon aphasia where words may be unrelated to the target, and those with no, or very little, expressive language or with apraxia of speech.

*You have to read. You have to read the text and say that’s the place. And then, yes, some people can't read.*

[P3]

*A lot of our patients that have jargon speech, some of it’s just non words. I don’t think that would be helpful for the AI if what they’re saying isn’t even real words [...] but even with real words a lot of it’s completely out of context, I think it’d be difficult for the AI to learn from that. So I think those patients should find that hard to use.*

[SLT5]

#### Other Neurological Impairments May Pose Challenges

Both groups reported that other stroke symptoms such as unilateral motor weakness, visual impairment, or cognitive difficulties affecting memory, self-monitoring, or initiation might make using R-SPEAK difficult or impossible.

*Yeah, some people, it’s disabilities, so they can't use their left arm or their right arm, and so they have to tap it.*

[P3]

*You need to be able to self-monitor the output and say whether it’s right or wrong and how much output there is. Can you cognitively cope with that burden?*

[SLT2]

#### Using Technology Is Easier for Some People

The people living with aphasia we interviewed found the tech quite easy themselves. Both groups thought that some people would be able to use R-SPEAK easily, perhaps those already using their smartphones and messaging apps. However, both groups thought that it might be more challenging for some who were less tech-savvy or not used to using smartphones.

*So probably people who were using phones to some extent before acquiring aphasia.*

[SLT4]

*I think it’s quite, quite easy to use and I think younger people will be even more easy to do use.*

[P1]

*And I think although he would really benefit from something like this because his speech, he’s hesitant. (But) he’s not confident with technology, neither would he have the right equipment.*

[C1]

#### Try Before You Buy

Given that some people may find R-SPEAK more difficult to use for a range of reasons and with potential cost implications, HCPs voiced the need for a “try before you buy” option.

*My first thought was cost [...] of a lot of the people I’m seeing, the first thing they think is actually they don’t want to pay for something until they know it’s worth it, and whether there’s a free trial [...] how long would people get to try it out? Because it’s quite rare that I come across people who are happy to pay for something straight away without trying it.*

[SLT3]

#### Some Training Needed

Both groups felt that people living with aphasia would need some initial training to use R-SPEAK; it might be something that an SLT could do, or perhaps a training video guide is built into the system.

*A family member or someone else could actually help somebody use the app potentially. It’s the sort of thing that somebody could help them with if needed.*

[SLT3]

*Speech therapist say [...] outlines the system. Yeah. And then full system, I do it with a guide.*

[P4]

### Perceived Usefulness of R-SPEAK

#### Improved Independence

Both people living with aphasia and HCPs thought that R-SPEAK could help improve independence.

*Well, (wife) has got she has me and her on the phone. She [...] regulates.*

[P4]

*He wants more freedom. He doesn’t want me having to sit here or go up to the reception desk or up to the bar or order a meal, which he does do because you know, he wants to. But sometimes people are getting confused, There’s a bit of embarrassment and stress levels are going up and how, you know, if you’re out for a meal, you don’t want to be. He wants to be able to do that himself. He’s 62.*

[C1]

*If this was a way of helping [PwA] get a bit more independence of going out and trying to communicate with strangers. Maybe that’s where it could come in handy.*

[SLT5]

#### Useful in Many Scenarios

Many ideas were generated about possible scenarios where R-SPEAK could be used. These included: between patients and care teams on wards, in restaurants, at the doctor’s surgery, during family conversations with young children, during phone calls or online meetings, and perhaps to support with writing emails or messaging. HCPs also suggested that R-SPEAK might be used therapeutically as a way to practice communicating.

*I know a lot of nursing staff sometimes struggle to understand what some with aphasia is saying, and it’s usually the weekend when the speech therapist isn’t there to try and interpret what it is they’re trying to say. So it could be useful If it’s helpful for them.*

[SLT5]

*I can imagine certain people sitting at home using it as a therapy tool, practising saying things, getting the feedback and choosing it and repeating after it, which is also another really useful use for it.*

[SLT3]

### System Design Features

People living with aphasia and HCPs liked that it was designed for use on a mobile phone, and they liked the interface due to its simplicity, font size, use of color, alignment, and spacing of features.

In terms of functionality, interviewees wanted to improve the accuracy and speed of the responses. Both groups made suggestions for improved functionality and suggested modifications including spoken responses for those who have difficulty reading, editable responses, an option to rerecord answers, or give 2 options to choose from. Table 4 summarizes the combined feedback on system design features.

**Table 4.** Combined feedback and suggestions for improvements on the design features of R-SPEAK^a^.

System design features	Positives	Negatives	Suggestions for modifications or improvements
Interface	Clear Simple Size and type of font Use of color Alignment of features On a mobile phone	No ability to modify	Include modifiable setup for: Language and accents Font size Bolding key words in text Color of background and text
Functionality	Accuracy sometimes good	Slow Accuracy sometimes poor	Improve accuracy of output Improve speed Include ability to hear response or read response Editable responses Rerecord option Recognize intonation of speaker, for example, statement versus question Present graphics instead of text output Learn from user to improve output Use information from the internet to improve output Available on all devices

a R-SPEAK: Revolutionizing Speech Enhancement in Aphasia Using Knowledgeable-AI.

### SUS

Ratings on the SUS by the participants with aphasia can be found in Figures 5 and 6. Individual SUS scores ranged between 62.5 and 85, with a mean score of 75 (SD 9.19), which overall demonstrates acceptability. Interviews lasted less than 1 hour and the focus group lasted 1.5 hours.

Given the small sample size, SUS and TAM findings should be interpreted as exploratory and indicative rather than confirmatory.

**Figure 5.**
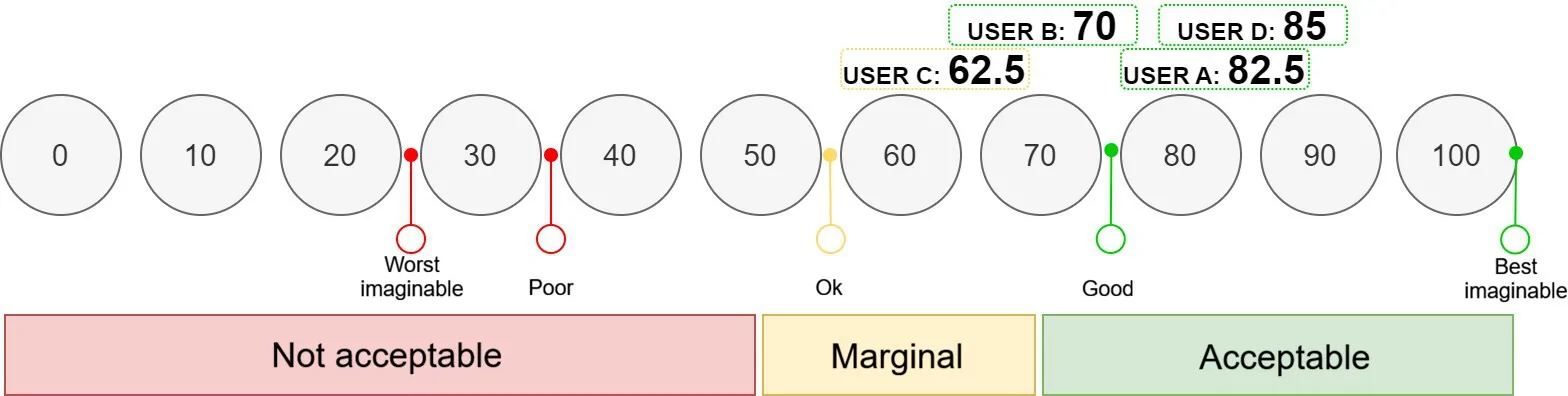
System Usability Scale (SUS): individual scores of participants with aphasia.

**Figure 6.**
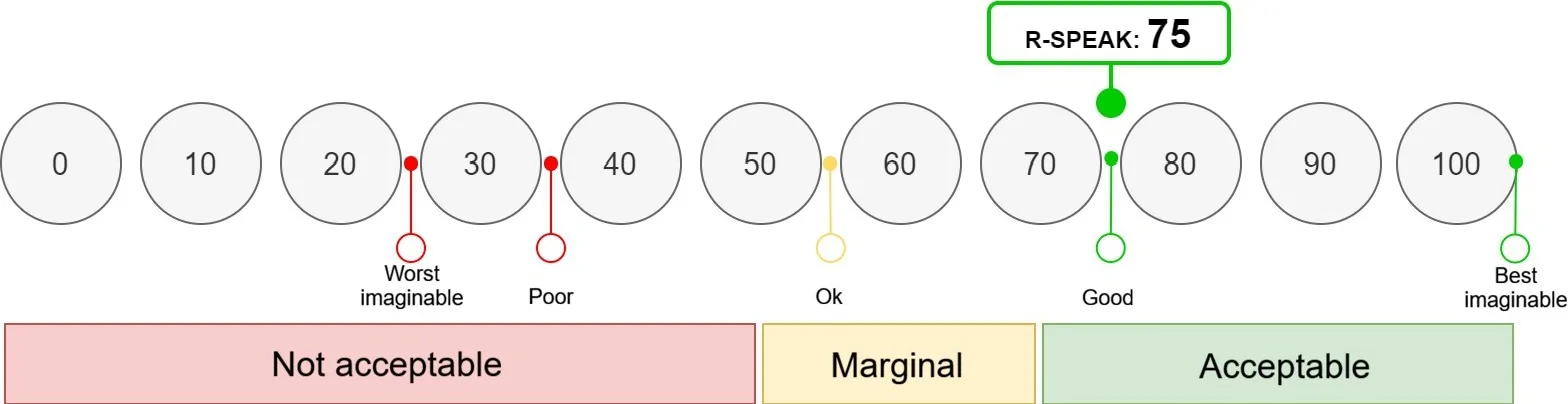
System Usability Scale (SUS): mean score of participants with aphasia.

### Phase 3: Refine and Redesign

#### Overview

In this phase, and in order to understand how well we can balance processing speed with clinical usefulness, we evaluated 12 open-weight lightweight LLMs on a dataset of 30 clinician-patient dialogue samples extracted from AphasiaBank. Validation analyses indicated that the LLM-as-a-judge approach produced consistent scoring patterns and demonstrated stable behavior across repeated evaluations.

#### Overall Performance and Model Rankings

The Qwen2.5:3B model emerged as the top performer overall, achieving an overall mean score of 0.814 (SD 0.046), as shown in Table 5. It attained 0.794 in relevancy, 0.866 in faithfulness, and 0.781 in completeness, with 100% pass rates across all samples and iterations. Hermes3:3B ranked second overall (0.796), excelling in answer relevancy (0.802, the highest among all models), though slightly trailing in faithfulness (0.826). Gemma3:4B placed third (0.792), demonstrating strong faithfulness (0.837) but showing higher variance (σ=0.184), likely due to sensitivity to iterative stochasticity.

**Table 5.** Performance comparison of tiny and small LLMs^a^ across key metrics.

Model	Answer relevancy	Faithfulness	Completeness	Mean score	Response time (s)
Qwen2.5:3B	0.794	0.866	0.781	0.814	0.600
Hermes3:3B	0.802	0.826	0.761	0.796	0.895
Gemma3:4B	0.787	0.837	0.753	0.792	0.738
Phi4-Mini:3.8B	0.779	0.796	0.741	0.772	0.815
Llama3.2:3B	0.777	0.777	0.743	0.766	0.704
Phi3:3.8B	0.776	0.761	0.735	0.757	0.800
Qwen2.5:1.5B	0.741	0.775	0.745	0.753	0.436
Gemma3:1B	0.679	0.806	0.753	0.746	0.451
Llama3.2:1B	0.670	0.772	0.707	0.716	0.487
SmolLM2:1.7B	0.700	0.759	0.678	0.712	0.425
DeepScaler:1.5B	0.607	0.712	0.668	0.662	5.162
Qwen2.5:0.5B	0.573	0.566	0.503	0.547	0.720

a LLM: large language model.

At the lower end, Qwen2.5:0.5B struggled across all dimensions (mean 0.547, SD 0.0386), with particularly poor faithfulness (0.566) and completeness (0.503), suggesting that sub-1B architectures lack sufficient capacity to reliably disambiguate fragmented aphasic input. DeepScaler:1.5B, while moderate in quality (0.662), exhibited prohibitively high latency (mean 5.16, SD 1.46 s), rendering it unsuitable for real-time assistive applications.

To contextualize the performance of lightweight models, we compared them against the top 3 performing larger language models used in Phase 1 of the study (namely, Mixtral:8x7B, Gemma2:9B, and Qwen2.5:7B). While these models achieved marginally higher overall scores (0.838, 0.833, and 0.807, respectively), this quality improvement comes at a disproportionate computational cost that could be prohibitive for interactive clinical deployment. Crucially, the performance gain over the best lightweight model (Qwen2.5:3B, 0.814) is minimal (+0.024 for Mixtral:8×7B), while inference latency increases dramatically from 0.60 to 2.42 seconds, representing a 4.0× slowdown that would disrupt the conversation flow.

This speed-performance profile is particularly significant for real-time clinical interactions. In therapeutic settings, response latency directly impacts conversational flow and patient engagement. Typical conversational gaps last only a few hundred milliseconds; silences of a second or more feel long and disrupt the flow. Individuals with communication disorders experience these prolonged pauses more often, increasing the effort needed to plan, produce, and process speech [34]. The Qwen2.5:3B model achieves near-optimal quality (within 3% of larger models), while maintaining subsecond response times essential for interactive apps. Even the fastest lightweight contender, SmolLM2:1.7B (0.43 seconds), offers clinically adequate performance (0.712 on average) at 5.6× the speed of the best larger model.

## Discussion

### Principal Results

We co-designed a prototype app using the Mixtral LLM to enhance the clarity and fluency of verbal expression of people living with aphasia. We tested the app with people living with aphasia and demonstrated it to HCPs. Both HCPs and people living with aphasia groups felt that R-SPEAK had the potential to be less stigmatized than other forms of AAC. As a mobile app, the software is more modern and can be integrated into the users’ existing devices compared to older AAC devices, which may lead to it being more socially accepted. Additionally, HCPs considered certain use-cases in which R-SPEAK would be useful, such as improving patient-doctor communication in clinical settings or even as a therapy tool.

The concept of practicing real-life communication as an intervention is not novel; speech therapy often involves role-playing in different scenarios, and EVA Park [35] is an online virtual world that gives people with aphasia an opportunity to practice communicating, but both examples rely on therapists to facilitate. In another study, people living with aphasia reported that the use of an app for communication practice would be highly beneficial for them [36]. R-SPEAK has the potential to be used in such a way. Both clinicians and people living with aphasia agreed that the potential use-cases for R-SPEAK could improve patients’ independence, lessening the need for SLT or carer intervention. This would remove some of the burden of the constant presence of carers and allow for people living with aphasia to gain more independence and confidence when it comes to communicating with others.

An average SUS score given by the people living with aphasia of 75 indicates a good level of usability, which is superior to other AAC apps for people living with aphasia, and the app’s interface was reported as easy to use. This implies R-SPEAK is acceptable to people living with aphasia. However, both groups expressed concern about the long response time of the prototype app. In the “Refine and Redesign” phase, we found that Qwen2.5:3B LLM achieved the strongest overall performance with high faithfulness and subsecond latency when compared to 11 other models.

There was a degree of disparity between the attitudes of people living with aphasia and HCPs; people living with aphasia were positive and expressed optimism about its potential to improve communication, whereas clinicians were more cautious about R-SPEAK’s capabilities to dramatically change the lives of people living with aphasia. HCPs doubted how well the tool would perform in actual conversation, where meaning can be highly contextual and implicative. Cost was also mentioned as a potential limitation to the software; charging people living with aphasia for a tool may deter use or even exacerbate the inequalities faced by people living with aphasia, particularly if they have idealistic expectations about the capabilities of R-SPEAK. A “try before you buy” feature was suggested to address this issue, to ensure that users are fully aware of the software’s capabilities before committing to payment.

Furthermore, concerns were expressed about the AI-powered nature of the app, which may deter potential users from engaging with the software; however, this sentiment was not shared by the people living with aphasia. People living with aphasia having higher expectations about the capabilities of R-SPEAK highlights a disparity between those with a specific lived experience of aphasia and those with purely clinical exposure. This conveys a potential struggle to align the expectations of the two groups. This observation highlights the crucial nature of co-design in this study for developing and evaluating clinical tools such as R-SPEAK. It is also essential to understand the important aims and outcomes of R-SPEAK for both the patient and the clinician for robust and meaningful testing.

While the participants of this study were able to use the software with ease, both groups had concerns about potential R-SPEAK users who were less technologically literate, had different types and severities of aphasia, or other stroke-related, speech, physical, cognitive, or visual impairments. This might limit the usability of the software and prompt more sophisticated (and therefore costly) training or personalization features. Therefore, incorporating a wider group of people with aphasia in further development and testing will be essential to ensure it is accessible to as many people as possible and so the target population could be refined. It was also suggested that HCP-provided training could be beneficial, although an in-app tutorial may be just as effective without the cost of having to formally train clinical staff.

### Limitations

R-SPEAK was developed specifically for patients with mild-to-moderate expressive aphasia; therefore, its effectiveness may vary for individuals with other types or severities of aphasia. However, given that 3 out of the 4 participants recruited to test the app had moderate aphasia and evidence of receptive aphasia but were able to use the technology with relative ease, this suggests that R-SPEAK could be adapted to suit patients with a wider range of needs. The participants had a moderate level of technological literacy, which might indicate that the positive attitudes and feedback may not be fully generalizable to those who are less technologically literate. In addition, during the testing, people living with aphasia were supported to use a web-based version of the app, which likely reduced the cognitive and linguistic demands on the participants and might mean feedback was overly optimistic [36]. This was a proof-of-concept study; thus participant numbers were intentionally small, but this is a limited representation of the target population and further testing on a wider group is imperative. Therefore, due to the modest sample size, the SUS and TAM results should be regarded as exploratory indicators rather than definitive evaluative outcomes. In future studies, more detailed demographic data such as gender, ethnicity, time living with aphasia, etiology, comorbidities, and so on would be beneficial to collect to understand how well the sample represents the population of people living with aphasia.

We also acknowledge that the sample size was insufficient to formally claim data saturation. However, participants were purposively selected for their extensive experience of the topic, resulting in highly information-rich data. Consistent with the principle of information power, the focused study aim, specificity of the sample, and depth of interviews supported the generation of credible findings. Furthermore, analytical rigor was enhanced through independent coding and team-based discussion of themes.

Another limitation stems from the initial testing of the LLMs, which involved evaluating their interpretation of utterances collected from AphasiaBank. The “gold standard” interpretations were decided upon by members of the team; however, it is difficult to confirm that these are in fact the true interpretations of the utterances because they were collected from unknown speakers out of the context of conversation. Moreover, the LLM used in the development of R-SPEAK was still prone to occasional underperformance, despite being rated the best LLM. Mixtral sometimes produced inaccurate interpretations of the patient’s utterance and overly wordy responses, which could lead to unproductive or confusing conversation turns. Further work in improving accuracy in outputs as misalignment between intended message and output reduces “trust” in AI.

### Comparison to Prior Work

R-SPEAK is a leap away from existing AAC tools, which typically require pointing to pictures or writing key words to express basic needs. More recent studies have started using AI to extend beyond traditional AAC methods. A US group [33] is codeveloping AI tools to support people living with aphasia in real-time communication and to prepare for future conversations. They used OpenAI’s DALL·E 3, ChatGPT 3.5 Turbo, and Whisper to build the tools. While it is not a complete product, the systems they are developing might be useful to consider as features in R-SPEAK, such as the double-checking of important words, using keywords to generate sentences, and the use of a diary to capture meaningful experiences. They are targeting a greater range of types and severities of aphasia, including those with comprehension difficulties, and they have included a range of participants in their prototype development. Participants reported that the tools reduced ambiguity and improved their ability to recount personal experiences with less effort, but trust in the AI tools was reduced due to technical limitations of AI.

Beyond this work, a growing body of research demonstrates the potential of AI and LLMs to support communication for people with aphasia. Early studies focused on sentence reconstruction using transformer-based language models trained on synthetic aphasic speech datasets, demonstrating promising improvements in grammaticality and sentence completion [37,38]. However, these studies were largely computational evaluations and did not provide real-time user-facing communication systems or incorporate user validation mechanisms. More recently, Aphasia-GPT explored the use of generative AI to transform impaired language into clearer utterances for AAC apps [39]. While promising, the study was limited to a pilot evaluation and did not integrate speech input, personalized voice output, or deployment considerations. Similarly, the same authors demonstrated that generative AI can reconstruct impaired language and improve communication effectiveness [40], but their work primarily focused on text-based reconstruction rather than complete conversational support.

Other emerging research has explored AI-enhanced AAC systems incorporating visual prompts, conversational assistance, and multimodal interaction [36,41]. These approaches highlight the value of AI-supported communication but often rely on concept selection, pictograms, or extensive user interaction rather than direct speech reconstruction. Reviews of AI-assisted aphasia technologies further identify challenges relating to trust, explainability, privacy, clinical validation, and deployment in everyday settings [42,43]. Notably, R-SPEAK was conceived and development commenced in September 2023, preceding several of these recent publications. R-SPEAK provides an end-to-end, user-controlled communication pipeline that combines ASR, LLM-based language understanding and completion, user verification, and personalized TTS within a modular architecture designed for local deployment, continuous improvement, and integration with existing assistive technologies.

### Suggestions for Future Work

The next step with R-SPEAK is to complete Phase 3 of the design process, the “Refine and Redesign” phase, in which design improvements are made based on feedback. Future testing may involve a larger sample of patients with a broader range of aphasia, confidence in technology, and other stroke-related impairments. This will ensure that the technology is usable by the entire target population, but also to observe whether patients outside of the initial target population might also benefit from R-SPEAK. Additionally, future testing may want to examine the software’s performance in real-world situations. This would involve testing the software in a specific context with an interlocutor, such as communicating patient needs in a hospital to a nurse.

Further work should also be done to improve the performance of the LLM technology for its application to R-SPEAK. Mixtral was found to underperform occasionally due to inaccuracy and its tendency to produce verbose outputs. Verbosity is a tendency of LLMs that have been trained on written text, since spoken language is nearly always more concise than written language. Future prototypes of R-SPEAK could leverage LLM technology by fine-tuning the model on speech data or using a model that has been pretrained on speech. This would prime the technology to produce more speech-like responses and therefore improve its performance.

The adaptable nature of LLM technology could be leveraged further to improve the user’s experience with R-SPEAK by offering features to tailor the software to the individual. For instance, the LLM model could be fine-tuned on the individual patient’s communication preferences, which would allow the model to learn their linguistic behavior and produce more accurate and personal responses. As mentioned by the people living with aphasia and HCPs in the focus groups, different patients may have specific difficulties; for example, they may struggle with reading comprehension or have motor problems. Implementing R-SPEAK as an experience tailored to the individual’s needs could both improve the general usability of the software and broaden the target population of R-SPEAK. For example, for those with motor impairments, a fully voice-controlled system might be more usable and therefore more beneficial. To develop and implement these features properly, further iterations of co-design and testing would be crucial to ensure that the software remains helpful and usable.

### Conclusions

This study introduces R-SPEAK, a pioneering solution that holds significant promise as a more effective and less stigmatized alternative to traditional AAC tools. Designed to enhance communication for people living with expressive aphasia (people living with aphasia), R-SPEAK leverages LLMs to provide a natural and engaging user experience. Initial testing revealed clinicians’ cautious optimism toward R-SPEAK’s impact and its generalizability, but people living with aphasia were enthusiastic and had high hopes for improving their lives. Cost and usability were main concerns; however, opportunities for improvement such as model enhancements, personalization features, and training materials pose opportunities to make R-SPEAK better-performing and more accessible to a wider range of patients.

## Abbreviations

AAC: augmentative and alternative communication
ASR: automatic speech recognition
CHAT: Codes for the Human Analysis of Transcripts
COREQ: Consolidated Criteria for Reporting Qualitative Research
HCP: health care professional
LLM: large language model
MoSCoW: Must Have, Should Have, Could Have, Will Not Have
NLP: natural language processing
PPI: patient and public involvement
R-SPEAK: Revolutionizing Speech Enhancement in Aphasia Using Knowledgeable-AI
SLT: speech and language therapist
SUS: System Usability Scale
TAM: technology acceptance model
TTS: text-to-speech
